# Autophagy in Prostate Cancer and Androgen Suppression Therapy

**DOI:** 10.3390/ijms140612090

**Published:** 2013-06-06

**Authors:** Elio Ziparo, Simonetta Petrungaro, Elettra Sara Marini, Donatella Starace, Silvia Conti, Antonio Facchiano, Antonio Filippini, Claudia Giampietri

**Affiliations:** 1 Institute Pasteur Fondazione Cenci Bolognetti, Department of Anatomy, Histology, Forensic Medicine and Orthopaedics (DAHFMO), Section of Histology & Medical Embryology, Sapienza University of Rome, Rome 00161, Italy; E-Mails: elio.ziparo@uniroma1.it (E.Z.); simonetta.petrungaro@uniroma1.it (S.P.); elettra.marini@uniroma1.it (E.S.M.); donatella.starace@uniroma1.it (D.S.); silvia.conti@uniroma1.it (S.C.); antonio.filippini@uniroma1.it (A.F.); 2 Laboratory of Applied Dermatology, Istituto Dermopatico dell’Immacolata, IDI-IRCCS, Rome 00167, Italy; E-Mail: a.facchiano@idi.it

**Keywords:** autophagy, apoptosis, prostate cancer, androgen deprivation therapy

## Abstract

The role of autophagy is known to be highly complex and context-dependent, leading to both cancer suppression and progression in several tumors including melanoma, breast and prostate cancer. In the present review, recent advances in an understanding of the involvement of autophagy in prostate cancer treatment are described. The regulatory effects of androgens on prostate cancer cell autophagy are particularly discussed in order to highlight the effects of autophagy modulation during androgen deprivation. A critical evaluation of the studies examined in the present review suggests the attractive possibility of autophagy inhibition combined with hormonal therapy as a promising approach for prostate cancer treatment.

## 1. Prostate Cancer and Therapy

Prostate cancer is one of the leading causes of cancer-related death in human males worldwide, being the second most current form of cancer in men. Prostate cancer grows locally at a slow rate, often for many years; eventually, it extends outside the prostate into neighboring tissues or by travelling to distant tissues through the blood. Common classification of prostate cancer stages is based on the TNM (tumor, nodes, metastasis) system thus indicating the size of the main area of prostate cancer, the involvement of lymph nodes and metastatic dissemination. The TNM system may be combined with the tumor grading of prostate cancer by the Gleason scoring system, usually ranging from 2 to 10 [1].

Locally advanced prostate cancer is initially hormone-sensitive and is treated with hormone therapy, also called androgen-deprivation therapy (ADT) or androgen suppression therapy. Approximately 50% of patients in industrialized nations are treated with ADT [2]. The main androgens are testosterone and the more active metabolite dihydrotestosterone. Such hormones are mainly produced in the testes and stimulate prostate cancer cells growth. Androgens act through binding and transactivating the androgen receptor (AR), which regulates gene expression by interacting with different co-regulators during prostate cancer progression. Lowering androgen levels, or preventing them from getting into prostate cancer cells makes prostate cancers grow more slowly. Several hormone therapy protocols can be used to support this aim. ADT can be accomplished with surgical castration (bilateral orchiectomy) or medical castration with luteinizing hormone-releasing hormone (LHRH) agonist therapy. LHRH agonists, by markedly reducing circulating testosterone level to so-called “castrate levels” (<0.5 ng/mL) over a sustained time, improve disease-free phase and overall survival, when used in combination with primary radiation for locally advanced or high-risk localized disease. Hormone therapy also includes “anti-androgen” therapy which is based on drugs blocking the action of these hormones. The use of “anti-androgens” is often combined with orchiectomy or LHRH analogues as first-line hormone therapy, called “combined androgen blockade” (CAB). To avoid a surge of testosterone in the first days of treatment by LHRH analogues, antagonists of LHRH have recently emerged as alternatives over the past few years [3]. Despite early success in suppressing prostate tumor growth, ADT ultimately fails, leading to recurrent tumor growth in a hormone-refractory (androgen-independent) state and the disease becomes castration-resistant (castration-resistant prostate cancer, CRPC). When patients with CRPC show symptoms of metastasis, cytotoxic chemotherapy is generally initiated (for a review see [4]).

When prostate cancer recurrence is observed, cancer cells are no longer responding to ADT even though they still express ARs [5]. ARs are expressed throughout prostate cancer progression and AR mutations, often found in hormone-refractory prostate cancers, allow AR-dependent transcriptional activity despite administration of targeted therapies designed to inhibit the receptor function [6]. Prostate cancer cells can “escape” from ADT, survive and develop androgen independence by several mechanisms. Cell survival mechanisms, allowing cancer cells to evade the death inducing process, consist in stimulation of growth factor pathways, activation of stress-dependent survival genes and enhancement of cytoprotective chaperone networks [7–9]. The autophagy process during androgen deprivation is also emerging in the literature as an additional way to escape hormone removal; this issue is described in detail below.

## 2. Autophagy

**Figure 1:**
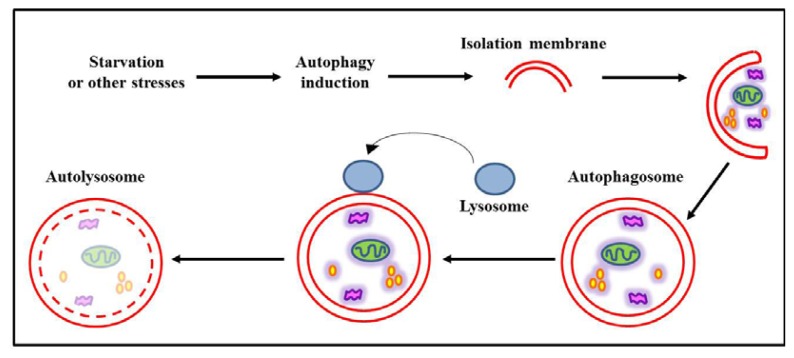
Schematic representation of autophagy. Autophagy starts when a membrane arises within a cell and engulfs cellular components leading to the creation of the autophagosome. It then fuses with a lysosome, thus forming the autolysosome, where cell components are degraded by lysosomal enzymes.

Autophagy is a genetically programmed, evolutionarily conserved process occurring in all eukaryotic cells. This dynamic process, schematically represented in Figure 1, mediates the clearance of long-lived cellular proteins and organelles leading to the formation of intracellular, double-membrane structures called autophagosomes, which sequester cytoplasm portions, proteins and organelles to break down and recycle materials [10]. Autophagosome formation is a multistep process involving several “autophagy-related proteins” (Atg proteins) and two ubiquitin-like conjugation systems, leading to the docking and fusion of autophagosomes with lysosomes to form the autolysosomes, which degrade the contents by lysosomal hydrolases [11]. Under optimal growth conditions, cells maintain a low basal level of autophagy, which can be rapidly enhanced by various stimuli (e.g., nutrient starvation, hypoxia, cytokines). In addition to its basic role in cell homeostasis, autophagy has multiple physiological and patho-physiological functions, resulting in either cell adaptation and survival or, under certain conditions, cell death. As a matter of fact, autophagy regulation is altered in several pathological conditions, such as neurodegenerative diseases and cancer [12].

## 3. “Dual-Faced” Role of Autophagy in Cancer

Autophagy has a controversial and still not completely clarified role in cancer. Cancer cells tend to re-program their metabolism machinery to evade cell death. In this context, when the tumor microenviroment is hypoxic and poor in nutrients, autophagy may help cancer cells to adapt to changing conditions, preventing their apoptotic death [13]. However, in contrast with the cancer-promoting effect of autophagy, human tumors commonly display mutations in autophagy-regulating genes, suggesting also an anti-cancer role of autophagy [14,15]. To date, the gene encoding the pro-autophagic protein Beclin1 is frequently monoallelically lost in human breast, ovarian and prostate tumors, highlighting a specific role for this protein as tumor suppressor [16]. Accordingly, the tumor suppressor gene PTEN, commonly mutated in cancer cells, induces autophagy [17], whereas the oncogenic protein Bcl-2 directly interacts with Beclin1 to inhibit autophagy [18]. Altogether these data confirm the dual role of autophagy in tumorigenesis, suggesting that the autophagic process may also act as an anti-cancer player, by limiting tumor cell growth or reducing mutagenesis or other damage caused by reactive oxygen species [19].

**Figure 2:**
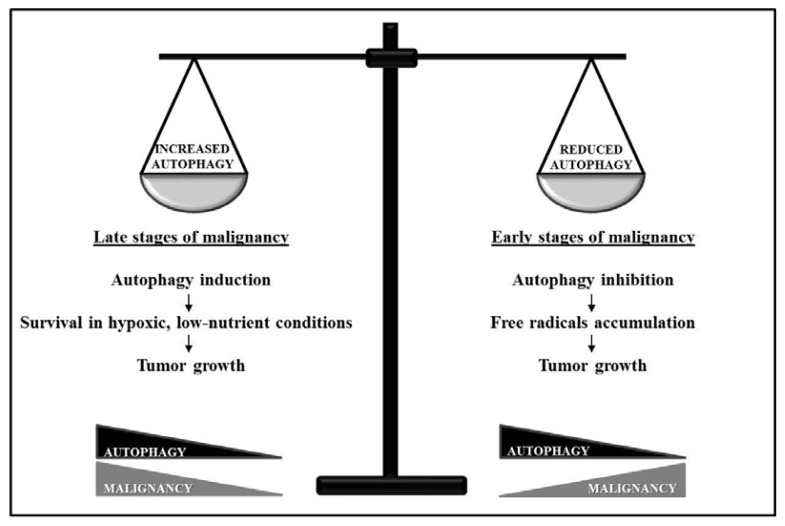
Autophagy role in cancer progression. The dual role of autophagy in cancer seems to depend on the stage of malignancy. At later stages autophagy induction represents a survival mechanism for cancer cells facing low-nutrient and hypoxic conditions. At earlier stages autophagy inhibition leads to malignancy increase allowing cancer cells to accumulate free radicals.

The controversial role of autophagy in tumor cell survival is also reflected in the ability of several anti-cancer treatments to exert an undesired cytoprotective effect by inducing autophagy, whereas others induce cell death through autophagy stimulation [14]. In particular such “dual-faced” role may vary according to the stage of the malignancy as represented in Figure 2. At the early stages, inhibition of autophagy sustains tumor growth by both limiting the rate of protein degradation and allowing accumulation of genotoxic free radicals [20]. Conversely, at later stages of tumor progression, autophagy is induced as a protective mechanism to allow cancer cells, located in the central areas of the tumor, to survive in the local low-nutrient and low-oxygen conditions [16]. Thus, it would be critical to define autophagy-regulating adjuvants for conventional therapies, in order to provide an efficient anticancer poly-therapy depending on treatment, cancer type, stage and context.

## 4. Autophagy in Prostate Cancer

The observation that *beclin1* gene is allelically deleted in many prostate cancers, suggested that autophagy might act as a tumor suppressor mechanism in the prostate [21,22]. Further studies performed in epithelial prostate cancer cells demonstrated that autophagy may also provide a survival mechanism to cells encountering stresses and therefore may represent a tumor promoting mechanism in prostate cancer [23]. Thus autophagy inhibition may be useful to make prostate cancer cells more sensitive to pro-apoptotic stimuli. It has been shown that an autophagy blockade sensitizes these cells toward Src tyrosine kinase inhibitor [24]. The Src kinase complex phosphorylates the androgen receptor, resulting in its nuclear translocation and activation; this kinase plays an important role in the development of castration-resistant disease state [25]. Indeed tyrosine kinase inhibitors targeting Src can inhibit androgen-independent growth of prostate cancer cells but do not induce significant apoptosis. In this context autophagy blockade significantly potentiates tyrosine kinase inhibitors pro-apoptotic effect [26].

In general the protective function of autophagy in cancer cells subjected to chemotherapy or radiation has led to generate intense interest in evaluating autophagy inhibition as a possible clinical strategy to counteract therapeutic resistance in prostate cancer [27–30]. On the other hand, in androgen-independent prostate cancer cells, it has been also shown that autophagy induction may sensitize cells to apoptotic stimuli [31,32] and radiation [33]. These data paradoxically suggest that, depending on the cellular features, either the induction or the inhibition of autophagy might provide therapeutic benefits to prostate cancer patients.

## 5. Androgen Deprivation Therapy (ADT) Stimulates an Autophagic Response

At least two mechanisms underlie the autophagy stimulation by androgen- ablation: the first one, shown *in vivo* in a mouse model, involves ADT-induced hypoxia in the tumor microenvironment, likely due to local vasculature degeneration [34]. Low oxygen induces autophagy in human tumor cells through multiple independent hypoxic pathways including HIF-1 transcription factor-mediated gene expression and mammalian target of rapamycin (mTOR) kinase inhibition. mTOR is a serine/threonine kinase composed of two main complexes. The rapamycin sensitive component is called mTOR complex 1 (mTORC1) which inhibits autophagy by directly phosphorylating and inactivating unc-51-like kinase 1 (ULK1), a proximal component of the autophagy signal transduction cascade. The second mechanism underlying the autophagy-induced by androgens absence has been demonstrated *in vitro* and involves multiple metabolic genes enhancing nutrient availability; in fact, since androgens normally activate such genes, prostate cancer cells, after androgen removal, face an energetic stress which turns on their autophagic response [35]. Autophagic vesicles, induced during androgen deprivation, may sequester lipid droplets, giving rise to the mechanism known as lipophagy. Since prostate cancer cells are rich in lipid droplets, lipophagy represents a key possibility to target their survival during ADT [36]. Furthermore, in the presence of energy deficiency caused by androgen removal, AMP-activated protein kinase (AMPK) is activated driving to suppression of mTOR signalling which promotes fatty acid oxidation, glycolysis [37] and autophagy [38]. Interestingly an *ex vivo* study has showed that AMPK is expressed at high levels in about 40% human prostate cancers thus confirming the frequent activation of a metabolic stress pathway [39]. The activation of AMPK is much stronger in androgen-independent than in androgen-dependent prostate cancer cells, leading to the hypothesis that cells with strong AMPK activation phenotype are better equipped for transition to androgen-independence [37].

Autophagy stimulation by androgen-ablation in prostate cancer cells parallels autophagy induction observed in breast cancer during anti-hormone therapies. Remarkably, as observed in prostate cancer, breast cancer cells also trigger autophagy to achieve a survival response that is critical for the development of anti-estrogen resistance [40].

## 6. A Possible Therapeutic Role for Autophagy in Prostate Cancer

Altogether the data presented in the scientific literature and reported above suggest metabolic stress-induced signalling pathways and autophagy as possible targets of intervention to inhibit androgen-independent prostate cancer development.

Autophagy inhibition may be therapeutically beneficial in various other cancers, as it can sensitize cancer cells to different therapies, including DNA-damaging agents, anti-hormone therapies (e.g., tamoxifen) and radiation therapy [23]. On the other hand, also autophagy stimulation by androgen removal may suggest autophagy blockade as a promising treatment during androgen deprivation therapy in prostate cancer. Studies performed *in vitro* on epithelial prostate cancer cell lines demonstrated that blocking autophagy by genetic and pharmacological means in the presence of androgen deprivation causes cell death. In particular chloroquine synergistically kills LNCaP cells during androgen deprivation in a dose- and time-dependent manner [36]. Similarly, pharmacological inhibition of autophagy enhances the efficacy of cell death mediated by androgen-ablation combined with chemotherapy [41]. Chloroquine and hydroxychloroquine, initially characterized as anti-malarial drugs by decreasing lysosomal function [42], appear to be promising cancer treatments [43]. Several clinical trials, conducted or in progress, have shown favorable effects of chloroquine as a novel antitumor drug [43,44]. In addition to chloroquine, other autophagy inhibitors, such as bafilomycin A1, 3-methyladenine, and pepstatin A, have been studied as antitumor drugs [45]. Unfortunately all such drugs are not specific modulators of autophagy activity and have other effects on cellular functions. Therefore, a more specific characterization of mechanisms involved in autophagy is essential for cancer therapy and is now under investigation [45]. Although *in vivo* studies on autophagy inhibition combined with androgen deprivation are still lacking, the results obtained *in vitro* sustain the potential therapeutic value of combining autophagy-modulation with conventional ADT *in vivo* for prostate cancer.

Autophagy inhibition during ADT might be useful to further sensitize prostate cancer cells to different apoptotic stimuli. In fact, prostate cancer cells evolve toward an androgen-resistant phenotype but they are still capable of undergoing apoptosis with appropriate stimuli [46]. Tumor necrosis factor-alpha (TNF-alpha) and TNF-related apoptosis-inducing ligand (TRAIL) are members of the death receptor ligand superfamily and have been suggested as potential anti-prostate cancer agents [47,48]. Remarkably, pharmacological autophagy inhibition is able to potentiate TNF-alpha-dependent apoptosis response in LNCaP cells [49–51]. Furthermore, blocking autophagy by pharmacological inhibitors or siRNAs targeting key autophagy factors Beclin1 or ATG7, effectively increases TRAIL-induced apoptotic cytotoxicity in prostate cancer cell lines [52]. Interestingly, autophagy blockade also potentiates cancer cell death induced by proteasome inhibitors [53]. Altogether such results obtained *in vitro* further indicate blocking autophagic process combined with targeted therapy as a promising therapeutic approach for prostate cancer.

Finally, findings regarding the cytosolic deacetylase HDAC6 role in autophagy progression strongly suggest that its inhibition may represent a promising novel basis for combinatorial treatments [54,55] in prostate cancer.

## 7. Autophagy Involvement in Modulating the Immune Response

Therapies impairing tumor metabolism modulating autophagy must consider their effect on lymphocytes activated in the immune response to cancer. The role of autophagy on anti-tumor immune cells has only recently begun to be defined [56,57]. It is not well understood to what extent autophagy contributes to reprogramming cellular metabolism in different immune cells experiencing stress and how this influences responsiveness to conventional anti-cancer agents. The immune system can mediate tumor eradication in some circumstances [58] and promote tumor growth in others [59]. Such different effects are the result of a complex mix of environmental factors. Antigen-presenting cells (APCs) of the innate immune system, such as macrophages and dendritic cells (DCs), play a central role in initiating anti-tumor immunity. Classically, dying tumor cells release soluble factors that promote phagocytosis by macrophages and DCs, resulting in APC-mediated priming of antigen-specific lymphocytes. Interestingly, autophagy has been demonstrated to be up-regulated at the immunological synapse during DC:T cell contact. Suppression of autophagy in DCs results in hyperstable contacts between the DC and CD4+ T cells and increases T-cell activation [60].

In the tumor microenvironment, several factors modulate cancer progression and severity [61]. Among different factors, Transforming Growth Factor-β (TGF-β) is one of the most studied, but its downstream signalling pathways are not fully understood. TGF-β is a multifunctional cytokine regulating cell growth, differentiation and apoptosis of various types of cells. TGF-β regulates different mechanisms influencing prostate homeostasis [62–66]. Its expression is elevated in most carcinomas and many proliferative diseases including benign prostatic hyperplasia, prostate cancer, and prostatitis [67–70]. TGF-β induces multiple effects on various signaling pathways leading to both tumor-inhibiting and -promoting actions. In normal tissues, for example, TGF-β signaling exerts anti-proliferative and apoptotic effects on epithelial cells. In contrast, in advanced cancers, TGF-β induces epithelial-to-mesenchymal transition, usually associated with cancer progression (for a review see [61]).

Autophagy has been recently described as one of the mechanisms activated by TGF-β, which leads to different effects on tumor progression in a cell-type-dependent and context-dependent fashion, but the relationship between TGF-β signaling and autophagy has not yet been clearly defined. It has been demonstrated that TGF-β activates autophagy in human hepatocellular carcinoma cell lines [71] and in some mammary carcinoma cells, indicating that autophagy induction is a novel aspect of biological function of TGF-β in cancer. Although the role of TGF-β–induced autophagy remains unclear, in early stages of carcinogenesis it may suppress tumor initiation in cooperation with other tumor suppressors, while in later stages of tumor progression, it might confer a growth advantage to cancer cells [61].

Interestingly, besides its role in tumor progression, TGF-β has been recently involved in ADT. Cross-talk between TGF-β and AR exists since androgens negatively regulate the expression of both TGF-β and its receptors *in vitro* [72,73]. Remarkably, TGF-β is over-expressed in human prostate tumors isolated from patients receiving ADT [74]. In light of these data, clarification of the molecular interactions between TGF-β and androgens signaling pathways is still an open question, an understanding of which may contribute to the design of new therapeutic approaches for prostate cancer treatment. It is tempting to speculate that TGF-β-induced autophagy may be one of the mechanisms involved in activating the resistance to ADT, in particular determining the ability to survive in an androgen-depleted environment by stimulating alternative survival pathways. Currently, TGF-β-signaling inhibitors have shown beneficial effects in clinical trials as anticancer agents (for review see [75]). Since autophagy activation might confer a growth advantage, it is conceivable to propose that the inhibition of TGF-β-signaling pathways during ADT might be a valuable therapeutic tool.

## 8. Perspectives

Continuous androgen receptor activation *in vitro* and *in vivo* has been also shown to cause cellular senescence, which is attenuated by androgen receptor antagonists [76]. Cellular senescence is a stable form of cell cycle arrest limiting the proliferation of damaged cells, thus cellular senescence may impose a potent barrier to tumorigenesis and contribute to the cytotoxicity of certain anti-cancer agents. It has been also demonstrated that autophagy is necessary to efficiently execute the senescence program [77]. Senescence is an early barrier to oncogenesis, thus the impairment of the efficiency or quality of senescence by autophagy inhibition may increase cancer incidence. In view of the above-described evidences, an understanding of the mechanisms linking autophagy and senescence in prostate cancer may be a novel field of investigation which should be considered in planning new therapeutic strategies based on autophagy modulation.

## 9. Conclusions

An interesting further point comes from the observation that, analyzing the 80 NIH-funded clinical trials currently recruiting prostate cancer patients more than 66 years old, (see Table 1 for the entire list), at least 50 (*i.e.*, almost 60%) are based on pharmacological treatments and interventions known to exert a known moderate effect (++) or a strong effect (+++) on autophagy. Table 1 also indicates the number of Medline abstracts reporting “autophagy” in “all fields”, for each of the treatments indicated. Table 1 again supports the modulation of autophagy as a promising therapeutic opportunity for prostate cancer patients.

**Figure 3:**
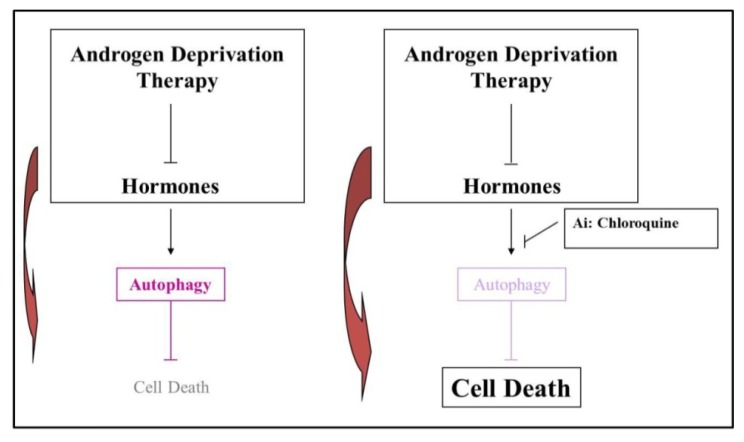
Inhibiting autophagy for prostate cancer therapy. Androgen deprivation therapy in prostate cancer cells induces autophagy as a resistance mechanism to elude cell death. Suppression of autophagy, using autophagy inhibitors (Ai), such as chloroquine, may potentiate cell death during androgen deprivation therapy.

Altogether data discussed in the present review highlight autophagy as an important process activated in prostate cancer after androgen removal; autophagy plays a relevant role for both cell physiology and immune system homeostasis and its inhibition combined with ADT must be considered as a valuable potential therapeutic opportunity to counteract prostate cancer growth by increasing cell death sensitivity (Figure 3). All these considerations make clear the urgent need of more potent and specific autophagic regulators as well as of a deep analysis of novel mechanisms controlling apoptosis-autophagy cross-talk.

## Figures and Tables

**Table 1:** Pharmacological treatments in 80 NIH-funded clinical trials currently recruiting prostate cancer patients.

NIH funded clinical trials	Interventions	Relation with autophagy	Number of Pubmed abstracts
1	Visualase Thermal Therapy System		
2	Ferumoxytol	++	1
3	Flutamide		
4	Abiraterone acetate; prednisone; veliparib	++ (prednisone; veliparib)	2 + 1
5	Tivantinib		
6	Metformin hydrochloride	+++ (metformin)	42
7	Radiation: fluorine F 18 sodium fluoride	+++ (radiation)	359
8	AMG 386; Abiraterone; Prednisone		
9	Sulforaphane glucosinolate capsules; capsules with cellulose and magnesium stearate	++ (sulphorane)	7
10	Akt inhibitor MK2206; bicalutamide	++ (Akt inhibitor)	5
11	Cabozantinib; Docetaxel; Prednisone	++ (docetaxel)	9
12	Akt inhibitor MK2206; hydroxychloroquine;	+++ (MK2206, hydroxychloroquine)	5 + 37
13	Finasteride	++ (finasteride)	1
14	Counseling intervention		
15	Bicalutamide; buserelin; flutamide; goserelin acetate; leuprolide acetate; orteronel; triptorelin	++ (bicalutamide)	3
16	Docetaxel; goserelin acetate; leuprolide acetate; surgery	++ (docetaxel)	9
17	Bicalutamide; buserelin; flutamide; goserelin acetate; leuprolide acetate; triptorelin; 3-dimensional conformal radiation therapy	++ (bicalutamide)	3
18	Radiation: radiation therapy; selective external radiation therapy	+++ (radiation)	359
19	Bicalutamide; goserelin acetate	++ (bicalutamide)	3
20	Radiation: 3-dimensional conformal radiation therapy; intensity-modulated radiation therapy; samarium Sm 153 lexidronam pentasodium	+++ (radiation)	359
21	Antiandrogen therapy; docetaxel	++ (docetaxel)	9
22	Abiraterone acetate; prednisone;	++ (prednisone)	2
23	Bicalutamide; flutamide; radiation therapy	+++ (bicalutamide; radiation therapy)	3 + 359
24	MR Imaging of the prostate using Amide-Proton-Transfer		
25	Genistein	++	9
26	Abiraterone acetate; degarelix; goserelin acetate; leuprolide acetate; orchiectomy		
27	Biological: Ad5-CMV-NIS; liothyronine sodium; iodine I 131	+++ (liothyronine, radiation)	2 + 359
28	Abiraterone acetate; dasatinib; prednisone	++ (dasatinib)	9
29	Hypofractionated radiation therapy	+++	359
30	Hydroxychloroquine	+++	37
31	Ipilimumab		
32	Cabazitaxel; prednisone; octreotide pamoate; octreotide acetate	++ (prednisone )	2
33	Axitinib; therapeutic conventional surgery		
34	Radiation: radiation therapy	+++ (radiation)	359
35	Oral L-arginine;	+++	35
36	Laser interstitial thermal therapy		
37	Oral microencapsulated diindolylmethane	++ (diindolylmethane)	3
38	Radiation: stereotactic body radiation therapy	+++ (radiation)	359
39	Lenalidomide; cyclophosphamide	++ (cyclophosphamide)	5
40	Abiraterone acetate		
41	Cinacalcet hydrochloride		
42	Motexafin gadolinium		
43	Radiation; Androgen Deprivation Therapy (ADT); L-BLP25	+++ (radiation)	359
44	Atorvastatin calcium	++	7
45	Transrectal prostate biopsy		
46	Docetaxel; pasireotide; prednisone	++ (docetaxel, prednisone)	9 + 2
47	Proton Beam Therapy; Intensity Modulated Radiation Therapy	+++ (radiation)	359
48	Dietary intervention; nutritional support	++ (dietary intervention)	5
49	Purified isoflavones; Methyl cellulose blend	++ (isoflavones)	21
50	Therapeutic conventional surgery		
51	Information Gathering;		
52	Proteomic profiling comprising MALDI-TOF MS,		
53	External beam radiation therapy; goserelin acetate	+++ (radiation)	359
54	Robot-assisted laparoscopic surgery		
55	Docetaxel; prostate biopsy; phenelzine sulfate	++ (docetaxel)	9
56	TNFerade™		
57	Radiation: brachytherapy; iodine I 125; palladium Pd 103	+++ (radiation)	359
58	Dietary Suppl. Se-methyl-seleno-L-cysteine; selenomethionine	++ (selenomethionine)	2
59	Behavioral: BF+GROUP; BF+PHONE		
60	Survey administration		
61	Gemcitabine; cisplatin; bevacizumab	+++ (gemcitabine; cisplatin)	13 + 103
62	Aerobic exercise	++	2
63	Memantine hydrochloride		
64	Texotere (Docetaxel); Alimta (Pemetrexed)	++ (Docetaxel, Pemetrexed)	9 + 3
65	NK cells +CliniMACs CD3 and CD56 systems	++ (CD3)	7
66	Lapatinib; paclitaxel	++ (Lapatinib; paclitaxel)	10 + 40
67	Radiation: radiation therapy; stereotactic radiosurgery	+++ (radiation)	359
68	Nicotine Replacement Patch	++ (nicotine)	3
69	Hyperthermia; Radiation: HDR brachytherapy	+++ (hyperthermia)	32
70	Behavioral: BF+GROUP; BF+PHONE		
71	Brachytherapy	+++ (radiation)	359
72	Veliparib	++	1
73	Behavioral: MR Therapy; Relaxing Music (RM) Therapy		
74	Radiation: Bone marrow sparing IMRT radiation therapy	+++ (radiation)	359
75	Polyphenon E;		
76	Selenium; vitamin E; selenium placebo	+++ (selenium; vitamin E)	13 + 26
77	Cabozantinib; FDG PET CT; NaF PET CT		
78	Biological: Autologous Ad HER2 dendritic cell vaccine		
79	Biological: recombinant albumin fusion protein sEphB4-HSA		
80	Behavioral: Home environs-based lifestyle counseling		

Search carried out at: http://clinicaltrials.gov/ct2/search/advanced; Search terms: prostate cancer; Status: recruiting; Study results: all studies; Study type: inteventional studies; Gender: male; Age: over 66 years; Phase: any; Funder: NIH.
